# Advances and gaps in SARS-CoV-2 infection models

**DOI:** 10.1371/journal.ppat.1010161

**Published:** 2022-01-13

**Authors:** César Muñoz-Fontela, Lina Widerspick, Randy A. Albrecht, Martin Beer, Miles W. Carroll, Emmie de Wit, Michael S. Diamond, William E. Dowling, Simon G. P. Funnell, Adolfo García-Sastre, Nora M. Gerhards, Rineke de Jong, Vincent J. Munster, Johan Neyts, Stanley Perlman, Douglas S. Reed, Juergen A. Richt, Ximena Riveros-Balta, Chad J. Roy, Francisco J. Salguero, Michael Schotsaert, Lauren M. Schwartz, Robert A. Seder, Joaquim Segalés, Seshadri S. Vasan, Ana María Henao-Restrepo, Dan H. Barouch

**Affiliations:** 1 Bernhard Nocht Institute for Tropical Medicine, Hamburg, Germany; 2 German Center for Infection Research (DZIF), Partner Site Hamburg-Lübeck-Borstel-Riems, Hamburg, Germany; 3 Department of Microbiology, Global Health and Emerging Pathogens Institute, Icahn School of Medicine at Mount Sinai, New York, New York, United States of America; 4 Friedrich-Loeffler-Institut, Institute of Diagnostic Virology, Greifswald-Insel Riems, Germany; 5 National Infection Service, Public Health England, Salisbury, United Kingdom; 6 Pandemic Sciences Centre, Nuffield Department of Medicine, Oxford University, United Kingdom; 7 Laboratory of Virology, National Institute of Allergy and Infectious Diseases, National Institutes of Health, Hamilton, Montana, United States of America; 8 Department of Medicine, Washington University School of Medicine, St. Louis, Missouri, United States of America; 9 Coalition for Epidemic Preparedness Innovations (CEPI), Washington, Washington, DC, United States of America; 10 Wageningen Bioveterinary Research, Lelystad, the Netherlands; 11 KU Leuven, Department of Microbiology and Immunology, Rega Institute for Medical Research, Laboratory of Virology and Chemotherapy, Leuven, Belgium; 12 Department of Microbiology and Immunology, University of Iowa, Iowa City, Iowa, United States of America; 13 Center for Vaccine Research and Department of Immunology, University of Pittsburgh, Pittsburgh, Pennsylvania, United States of America; 14 Kansas State University, College of Veterinary Medicine, Manhattan, Kansas, United States of America; 15 World Health Organization, Geneva, Switzerland; 16 Tulane National Primate Research Center, Covington, Louisiana, United States of America; 17 Vaccine Research Center, National Institute of Allergy and Infectious Diseases, National Institutes of Health, Bethesda, Maryland, United States of America; 18 Centre de Recerca en Sanitat Animal (CReSA, IRTA-UAB), Campus UAB, and Departament de Sanitat i Anatomia animals, Facultat de Veterinària, UAB, Barcelona, Spain; 19 Australian Centre for Disease Preparedness, CSIRO, Geelong, Australia; 20 Center for Virology and Vaccine Research, Beth Israel Deaconess Medical Center, Harvard Medical School, Boston, Massachusetts, United States of America; University of North Carolina at Chapel Hill School of Medicine, UNITED STATES

## Abstract

The global response to Coronavirus Disease 2019 (COVID-19) is now facing new challenges such as vaccine inequity and the emergence of SARS-CoV-2 variants of concern (VOCs). Preclinical models of disease, in particular animal models, are essential to investigate VOC pathogenesis, vaccine correlates of protection and postexposure therapies. Here, we provide an update from the World Health Organization (WHO) COVID-19 modeling expert group (WHO-COM) assembled by WHO, regarding advances in preclinical models. In particular, we discuss how animal model research is playing a key role to evaluate VOC virulence, transmission and immune escape, and how animal models are being refined to recapitulate COVID-19 demographic variables such as comorbidities and age.

In February of 2020, the World Health Organization (WHO) R&D Blueprint convened a group of experts to develop preclinical models of Severe Acute Respiratory Syndrome Coronavirus 2 (SARS-CoV-2) infection. Since its inception, the goal of this WHO COVID Modeling group (WHO-COM) has been to accelerate the development of Coronavirus Disease 2019 (COVID-19) vaccines and therapeutics by rapidly sharing data among member scientists worldwide. In addition, concerns were raised at that time about the possibility of vaccine-associated enhanced respiratory disease (VAERD) or antibody-dependent enhancement (ADE) after vaccination or infection. In September of 2020, the WHO-COM published a review on COVID-19 animal models [1], which reflected the state-of-the art at that time, with the vast majority of publications authored by members of the group.

Preclinical studies in nonhuman primates (NHPs) of COVID-19 vaccines that are currently being deployed [2–5] proved remarkably predictive of the outcome of clinical efficacy studies. In particular, NHP studies not only predicted high clinical efficacy of these vaccines but also suggested immune correlates of protection. Moreover, preclinical studies accurately predicted that protection against severe pneumonia would be easier to achieve than protection against viral replication in nasal mucosa. These observations confirm the value and importance of the use of animal models for COVID-19.

In 2021, with several vaccines rolling out worldwide and the detection of variants of concern (VOCs), the development of preclinical models of SARS-CoV-2 infection and their role in COVID-19 research has entered into a new phase. This paper provides an update from the WHO-COM regarding advances in preclinical models. In particular, we discuss how animal model research has provided insight into VOC pathogenesis and correlates of protection and has helped therapeutic development. Finally, we discuss the current status of VAERD research and the race to develop models that recapitulate COVID-19 demographic variables such as comorbidities and age.

## Animal models to study VOCs

As the current pandemic evolves, several virus variants carrying multiple mutations have emerged in different regions of the world. Some variants have been classified by WHO as variants of interest (VOIs) or VOCs, based on epidemiological evidence of enhanced transmission and possible evasion from natural and vaccine-induced immunity [6]. Animal models have a key role in the evaluation of VOC transmission, immune escape, and pathogenicity.

### Vaccine cross-protection and transmission

Recent studies in mice, hamsters, and NHPs show that animals previously infected or vaccinated against lineage A SARS-CoV-2 (for example, the original Wuhan strain) [7] are protected against challenge with homologous as well as heterologous virus strains including the alpha (B.1.1.7), beta (B.1.351), gamma (B.1.1.28.1), and delta (B.1.617.2) VOCs [8–14]. In the NHP model, however, more viral breakthroughs were observed following beta VOC challenge as compared with homologous WA1/2020 challenge [12,15]. In addition to protection against disease, another concern was to determine whether reinfection with VOCs resulted in SARS-CoV-2 shedding, which would raise the possibility that asymptomatic reinfected individuals might transmit VOCs. In this regard, hamsters reinfected with VOCs were indeed shown to shed SARS-CoV-2 for a number of days [8,14,16]. However, transmission studies performed in cats indicated that infected animals did not shed enough virus for transmission to cohoused naïve sentinel cats [17]. These results are in agreement with the finding that, although vaccinated individuals can be reinfected, transmission of delta VOC from these individuals may be substantially reduced in comparison with nonvaccinated subjects [18]. Importantly, virus shedding in hamsters, ferrets, and NHPs was reduced by intranasal vaccination [19,20], showing perhaps an added value of mucosal vaccines to control VOC expansion.

It is now clear, however, that, in competition studies, at least the alpha and beta VOCs show enhanced transmission in comparison with lineage A SARS-CoV-2 in a number of models, including hamsters, ferrets, and white-tailed deer [21–24]. This attribute could be dependent, at least partially, on the presence of VOC-specific spike substitutions such as N501Y, D614G, and V367F, which improve the affinity of the SARS-CoV-2 spike protein for the human and hamster angiotensin-converting enzyme 2 (ACE2) receptors. The aromatic N501Y substitution that is present in the alpha, beta, and gamma VOC is associated with increased transmission in humans but also allows for infection in the wild-type mouse using the mouse ACE2 receptor [25]. Thus, 2 recent studies have shown that wild-type mice are susceptible to certain SARS-CoV-2 VOCs, specifically to the beta (B.1.351) and gamma (P1) VOCs [26,27], most likely due to the N501Y substitution. Although mild lesions and viral replication were observed in nasal turbinates and lung of these wild-type mice inoculated with these VOCs, no significant clinical signs were observed. These results open the avenue to use wild-type mice as a potential animal model of asymptomatic infection with SARS-CoV-2, mainly to study immune responses, for which laboratory reagents are widely available. More threatening, these observations also raise concerns on the possibility of interspecies transmission, with new variants expanding their tropism toward other animal species resistant to the ancestral viral strains and, eventually, becoming novel secondary viral reservoirs [28]. However, the highly relevant delta variant has a different mutation pattern that does not include an N501Y substitution, but a P to R substitution in the spike protein cleavage site and therefore may react differently in the animal models than other VOCs. This raises the possibility that SARS-CoV-2 could also evolve into a human-specific virus with reduced cross-infective properties in other mammals. In this regard, human tissue culture microfluidic systems have been developed to supplement the infection modeling landscape [29,30] and may offer a scalable solution to such a scenario if it were to emerge. Characterization in the full spectrum of available models and systems and with different delta VOC isolates is therefore suggested. However, it must always be kept in mind that the emergence of additional new VOCs will lead to a time delay in testing in animal models, since the selected viruses must first be isolated and characterized in vitro and subsequently distributed to the different laboratories worldwide.

### VOC pathogenesis in animal models

**Fig 1:**
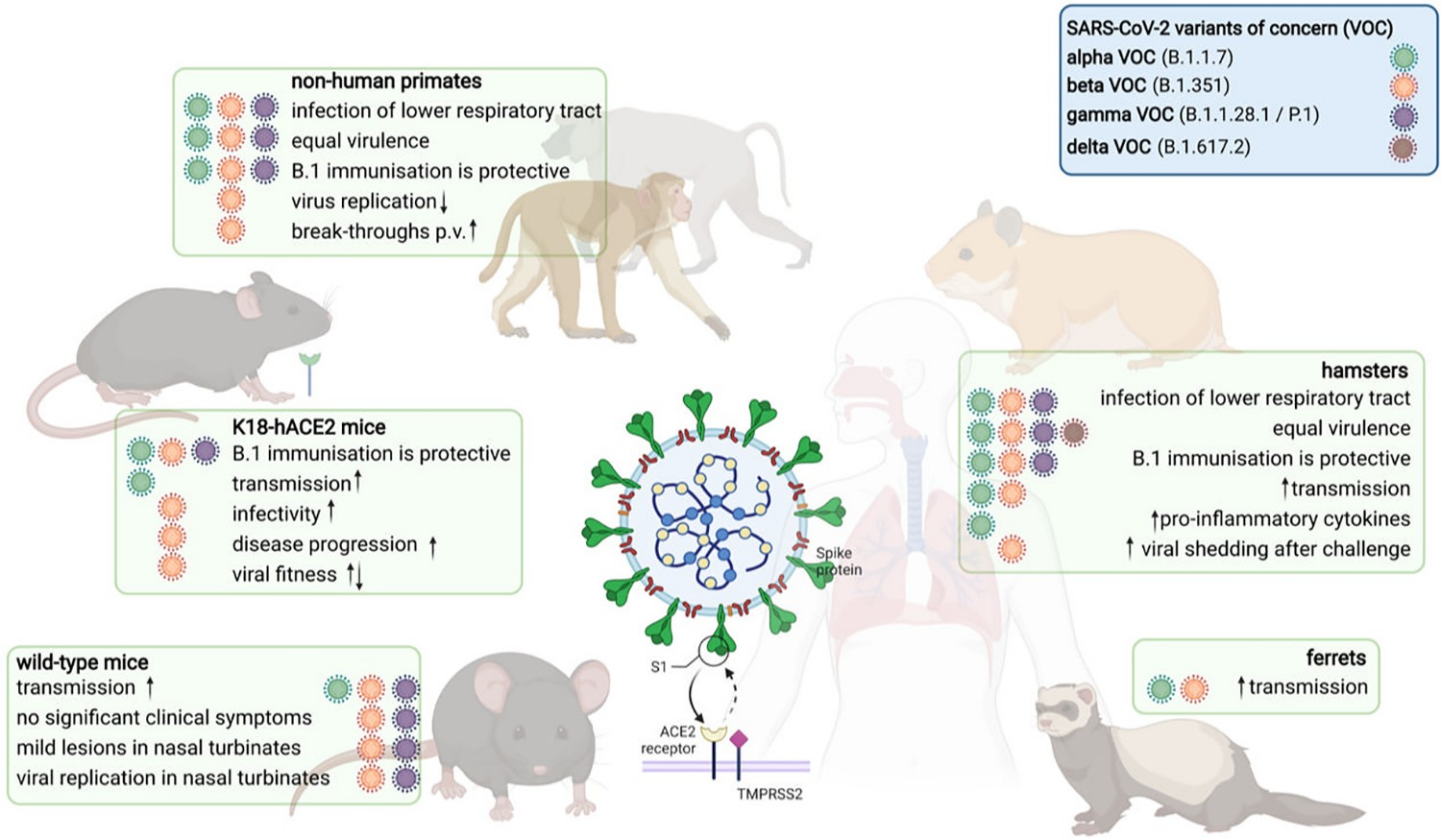
SARS-CoV-2 VOC in animal models. The schematic summarizes findings related with virulence, transmission, and cross-protection gathered in the indicated animal models so far. Figure created with Biorender (Biorender.com). huACE2, human angiotensin I-converting enzyme 2; K18, cytokeratin-18; SARS-CoV-2, Severe Acute Respiratory Syndrome Coronavirus 2; VOC, variant of concern.

Importantly, even though VOCs readily infected the lower respiratory tract of hamsters and NHPs, none of these variants seemed to show enhanced virulence in these animal models, although increased production of proinflammatory cytokines was observed in hamsters infected with the alpha VOC [9]. In a comparative study carried out in rhesus macaques, infection with the beta VOC resulted in lower clinical scores and lower levels of virus replication in comparison with ancestral B.1 virus and alpha VOC [31]. These findings were confirmed in a direct comparison between B.1 and alpha VOC infection in African green monkeys [32]. Conversely, studies in other models such as transgenic mice expressing the human ACE2 (huACE2) receptor driven by the cytokeratin-18 (K18) gene promoter (K18-huACE2) have yielded different outcomes. In these mice, infection with the beta VOC resulted in enhanced infectivity and a quicker disease progression in comparison to one of ancestral variants (B.1) [26]. Such enhanced infectivity could be due, at least to some extent, to the expression of higher levels of interferon antagonist proteins by some VOCs [33]. Other studies have, however, shown reduced fitness of the beta VOC in mice in competition trials [22] (Fig 1). One possible explanation for differences in VOC virulence when comparing results in different animal models is the rise of mutations leading to increased processing and fusion by the S protein of SARS-CoV-2 [34,35]. As VOC phenotypic features appear dependent on interactions with host factors, experimental demonstration of enhanced virulence or transmission with emerging VOC may depend on the animal model and the specific VOC strain used. One strategy to minimize the variables during experiments has been the use of syngeneic viral backbones generated by reverse genetics [36]. This approach demonstrated the fitness advantage of S-614G versus S-614D in a syngeneic direct competition assay [21] and recently also demonstrated the role of the spike protein of the alpha VOC for enhanced transmission properties in a transgenic mouse model [22]. It is important to consider that various experimental setups and the use of different VOCs could affect the experimental outcome. A series of experiments in different animal models and with different VOCs are necessary for particularly robust conclusions.

## Vaccine-associated enhanced respiratory disease

Vaccine-associated enhanced disease (VAED) defines adverse events that affect individuals infected with a pathogen after receiving a prior vaccine against that same pathogen. More specifically, VAERD was observed in individuals immunized with formalin-inactivated vaccines against measles or respiratory syncytial virus (RSV) decades ago [37,38]. ADE, on the other hand, is a phenomenon where preexisting vaccine or infection-induced antibodies enhance infection of FcγR expressing target cells, which results in increased disease. ADE is well documented to occur after secondary infections with dengue viruses belonging to different serotypes [39].

There were initial concerns that SARS-CoV-2 vaccines, in particular whole-inactivated vaccines, might lead to VAERD or ADE. These concerns were heightened by in vitro evidence of ADE for SARS-CoV and Middle East Respiratory Syndrome (MERS)-CoV [40], experimental data indicating eosinophilic VAERD in studies of SARS-CoV and MERS-CoV vaccines in mice [41,42], and hepatitis observed in a SARS-CoV ferret model [43]. These concerns led to the assessment for possible VAERD and ADE by the WHO-COM group in different animal models of SARS-CoV-2 pathogenesis.

These initial concerns have been now alleviated by several findings in animal models. First, all vaccines that either are emergency use authorized or in late clinical development phases induce strong T-helper 1 (Th1) CD4-mediated responses, which are associated with high ratios of neutralizing versus nonneutralizing antibodies and reduced risk of VAERD [44]. Moreover, several experiments performed by WHO-COM scientists have utilized experimental alum-adjuvanted and formaldehyde-inactivated whole virus vaccines and subsequent SARS-CoV-2 challenge to address any evidence of VAERD. In rhesus macaques, histopathological analysis revealed no evidence of enhanced lung pathology, and a rapid rise in neutralizing antibodies was seen after challenge [45]. In hamsters and ferrets, on the other hand, a mild increase of lung pathology was observed at 5 days and 7 days postinoculation (dpi), respectively, in comparison to unvaccinated controls. Nonetheless, in both models, pathology was reduced at 13 dpi to comparable levels for vaccinated and unvaccinated animals. The enhanced pathology was characterized by increased perivascular cuffing (ferrets and hamsters) and greater influx of mononuclear cells and granulocytes in alveoli with thickening of alveolar wall, proliferation of type II pneumocytes, and hemorrhages (hamsters). No clear influx of eosinophils was observed in either species. Noteworthy, hamsters showed no neutralizing antibodies post-immunization and no protection against challenge, but lung cytokines were markedly skewed toward Th2 [45]. In addition, a recent study in K18-hACE2 mice immunized with a very impure formalin-inactivated SARS-CoV-2 preparation and an aluminum hydroxide-based adjuvant demonstrated earlier onset of SARS-CoV-2 replication and disease in comparison to the naïve control groups or mRNA-vaccinated animals [46].

As VAERD typically develops after vaccine-induced antibody responses wane, it may be too soon to conclude about the presence of VAERD in SARS-CoV-2 vaccination. However, the data currently available from animal experiments, showing the absence of VAERD in NHPs and a transient increase of lung pathology in ferrets and hamsters, are reassuring. Moreover, VAERD has not been reported in humans immunized with inactivated SARS-CoV-2 vaccine preparations.

## Animal models and VOC prophylaxis and therapy

Despite the rapid and successful development of COVID-19 vaccines, unequal global vaccine distribution has contributed to the rise of VOCs with potential to escape natural as well as vaccine-induced immunity. This, together with the lack of medical countermeasures against severe COVID-19, has resulted in increased efforts to develop prophylactic and therapeutic strategies against SARS-CoV-2 infection, for which animal models are playing a key role. Although revising all therapeutic strategies against COVID-19 is out of the scope of this study, we would like to point out recent preclinical studies with potential to treat infections caused by VOCs. Of these, the use of polyclonal antibodies or antibody cocktails against the SARS-CoV-2 spike receptor binding domain (RBD) provide an advantage by binding to multiple epitopes, thereby reducing the chance of VOC immune escape. Such strategies have shown potent prophylactic and therapeutic activity in mouse models of infection as well as hamsters [47,48]. Alternatively, monoclonal antibodies such as COVA1-18 and P5C3 with broad neutralizing activities have also shown prophylactic effect in hamsters, hACE2 mice, and cynomolgus macaques and potent reduction of virus replication postexposure [49,50]. P5C3 has also shown neutralization of all known VOCs to date at picomolar concentrations [50]. Neutralizing single domain antibodies (nanobodies) can be administered intranasally and have also shown to greatly reduce SARS-CoV-2 replication in hamsters [51,52]. A complementary strategy against VOC could be the development of antibody cocktails with enhanced Fc-mediated functions, which have been shown to greatly contribute to SARS-CoV-2 humoral immunity [53]. Consequently, anti-SARS-CoV-2 monoclonal antibodies with optimized Fc domains have shown potent prophylactic and therapeutic activity in several mouse models and hamsters [54].

In the early days of the pandemic, animal models were also key for testing nucleoside analog drugs such as remdesivir [55] and favipiravir [56]. More recently, molnupiravir (MK-4482) has shown great potential as an orally available drug, which significantly reduced SARS-CoV-2 replication in hamsters [57] and prevented transmission in ferrets [58]. Nucleoside analogs such as favipiravir have also shown synergistic activity in combination with other drugs such as 3CL protease inhibitors in huACE2 mice [59]. Thus, animal models may be needed to test combination drugs against COVID-19. For this, as we will discuss below, the development of more severe models of disease may be needed.

## The search for an animal model of severe SARS-CoV-2 disease

One of the main gaps still remaining in the development of SARS-CoV-2 infection models is the identification of a preclinical animal model that recapitulates the severe and lethal form of human COVID-19. Such a model would be of great advantage for several aspects of research. First, it would provide a tool to study the transition from mild to severe disease, which would possibly lead to the identification of disease mechanisms and biomarkers. Second, it would expand the presently available animal models to evaluate vaccines and therapeutics, which could result in urgently needed rescue medical countermeasures. Since the first review published by the WHO-COM [1], the hamster model has emerged as the one that more closely recapitulates moderate disease in humans. Hamsters not only develop respiratory disease after SARS-CoV-2 infection, but also display some other important clinical hallmarks in patients such as anosmia, neurotropism, and vascular inflammation [60–62]. With some exceptions, most laboratories have observed that hamsters tend to lose weight rapidly after experimental infection with SARS-CoV-2 reaching 5% to 20% or more of body weight loss at the peak of infection. However, in some parts of the world, ethical approvals establish euthanasia endpoints at 20% to 25% of weight loss, while in other laboratories, animals are sometimes allowed to lose 30% of body weight. These regulatory differences complicate the definition of severe disease in animal models of infection. Nevertheless, pathological examination of infected hamster lungs shows evidence of severe interstitial pneumonia with high levels of bronchoalveolar damage and inflammation. Unfortunately, the hamster model presents a few unresolved challenges; for example, there is a need to better understand the relationship between the severity of pulmonary pathology and the mild to moderate clinical signs. In order to address this, it seems imperative to develop more tools to study hamster immunology, for example, antibody panels for multiparametric flow cytometry. Moreover, male hamsters show more severe lung lesions than females and less efficient antibody responses [63,64]. In the absence of comorbidities or coinfections, an explanation for these findings is likely dependent on sex-associated differences in immune responses. Indeed, male COVID-19 patients have shown higher levels of proinflammatory cytokines and reduced T cell–mediated immunity in comparison with female patients [65]. Whether this is the case also in the hamster model again depends on the development of more advanced tools to study hamster immunology.

Furthermore, the disease development may also be related to age of the used hamsters with older hamsters showing more severe disease progression [66], although others found no substantial age-related difference [67,68]. Despite the gaps of knowledge, hamsters have become the model of choice for preclinical testing of vaccines and therapeutics together with different species of NHPs [69–72].

In addition to the hamster model, several murine models of severe disease are now available, including infection of common laboratory strains with mouse-adapted SARS-CoV-2 and certain SARS-CoV-2 VOCs and infection of mice expressing huACE2 transgenically or of mice with “knock-in” of huACE2 [73–76]. Mice develop pathological signs of pneumonia that range from mild to severe. In some instances, mice also develop anosmia, a common manifestation of the human COVID-19 [76]. Mice have the advantage of well-characterized genetics and the availability of mice that are completely or conditionally deleted in genes of interest. In addition, the vast existence of reagents to study immune responses in mice also allows a much better characterization of the immunology related to SARS-CoV-2 compared to hamsters or ferrets.

Finally, as discussed above, several monoclonal antibodies and small molecule antivirals against SARS-CoV2 [9] are in development, some of which are approved in the United States or may receive market authorization elsewhere in 2021. An important open question is whether escape or resistant virus variants may emerge against either of these therapies. The current SARS-CoV2 animal infection models typically show a limited duration of virus replication and are therefore not well suited to explore whether, in particular with suboptimal doses of either a monoclonal antibody or a small molecule antiviral, drug-resistant variants may emerge. It will therefore be important to develop SARS-CoV2 infection models in which the virus replicates to sufficiently high titers for extended periods of time without causing severe pathology that would require early euthanasia of these animals. To this end, either strains of animals with immunodeficiencies or the experimental induction of immunodeficiencies by pharmacological interventions would be worth exploring.

## Age and comorbidities

In human COVID-19 disease, there is a strong association of severe disease with age and/or preexisting comorbidities including cardiac disease, diabetes/obesity, hypertension, and chronic respiratory diseases. For COVID-19, age is the best correlate of poor outcome, with those over the age of 85 having a 630-fold increase in death compared to those 18 to 29 years old (https://www.cdc.gov/coronavirus/2019-ncov/covid-data/investigations-discovery/hospitalization-death-by-age.html). Refinement of preclinical models to recapitulate the effect of age and comorbidities in SARS-CoV-2 infection has been an important effort during the last year.

### Age

Mouse models were one of the first options to explore the effect of age in SARS-CoV-2 infection. There are numerous mouse models of aging that could be applied to COVID-19 studies. The main strategy has been to cross these mouse models with huACE2 transgenic mice or to use mouse-adapted virus. Overall, these studies indicated that the severity of SARS-CoV-2 infection in C57BL/6 and Balb/c mice is age dependent [73]. Indeed, young mice were resistant to SARS-CoV infection even if they lacked IFN-I expression or the capacity to mount adaptive immune responses [77], while aged mice infected with SARS-CoV-2 showed greater weight loss, clinical signs, and pathology than their young counterparts despite comparable viral loads [73]. Mechanistically, the positive correlation between age and severity in SARS-CoV–infected mice was associated with increased age-dependent inflammation in the lung [78], which is consistent with findings in humans [79].

The effect of age in SARS-CoV-2 infection has been also evaluated in ferrets. These studies showed that 1- to 2-year-old ferrets had prolonged fever compared to young ferrets, which developed little-to-no fever. Three-year-old ferrets had fevers that lasted out past 10 dpi. In addition, older ferrets lost more weight upon infection and regained it more slowly than younger ferrets [80]. More severe lung pathology also was noted in the older ferrets at 5 dpi. Of note, older ferrets also had higher viral titers in nasal washes and fecal specimens for a longer period and were more likely to transmit the virus to younger ferrets. This is also consistent with findings in older patients, in which immune senescence, loss of type I IFN responses, decline in antigen presentation, and reduced T cell responses have shown to delay viral clearance [81,82].

This correlation between SARS-CoV-2 infection severity and age has been also confirmed in the NHP model. A study comparing rhesus macaques and baboons suggested that viral pneumonia may persist longer in older animals of either species, and a reduction in antibody responses in aged rhesus macaques [83]. Aged rhesus macaques also have been shown to have increased rectal shedding, slower viral clearance, higher viral loads, more severe lung pathology, higher levels of proinflammatory cytokines, and greater body weight loss [84]. Increased shedding of viral RNA from the upper respiratory tract was observed in older cynomolgus macaques in one study, but this was not associated with increased disease severity [84]. A multiomics study comparing subadult and aged rhesus macaques showed that age did not substantially affect acute disease; however, an age-specific divergence of immune responses emerged during the postacute phase of infection (7 to 21 dpi). As in humans, advanced age resulted in a delayed or impaired induction of antiviral cellular immune responses and a delay in the efficient return to immune homeostasis [85].

### Comorbidities

Less is known about the effect of comorbidities during SARS-CoV-2 infection in animal models. The reason is that, with the exception of mice, comorbidities are difficult to model in animal experiments. In mice, however, the huACE2 model can be crossed with other specific models of disease such as diabetes, chronic inflammation, or cardiovascular disease (CDs) models. Alternatively, mouse-adapted SARS-CoV-2 or any of the VOCs containing the N501Y spike protein mutation can be directly used to infect mice. Finally, huACE2 can be also expressed to mouse models of disease via adenovirus delivery. The latter approach has been used to study the effects of CDs and diabetes mellitus in SARS-CoV-2 infection and has shown that preexisting CDs resulted in enhanced inflammation and risk of myocardial injury upon infection [86], which is consistent with observations in humans [87]. Another strategy is the induction of comorbidities, in particular diabetes and obesity, through changes in rodent diet. Thus, diet-induced obesity in mice resulted in more severe disease upon infection with N501Y-containing SARS-CoV-2 [25]. Similarly, hamsters fed a western diet for 16 weeks showed greater weight loss and higher viral loads, as well as prolonged viral shedding compared to controls fed a regular rodent diet [88]. Despite these differences, there were no significant effects of obesity in respiratory function or SARS-CoV-2–induced pathology between the 2 groups. Other comorbidities associated with severe disease in COVID-19 have not yet been studied in rodents.

## Correlates of protection

**Fig 2:**
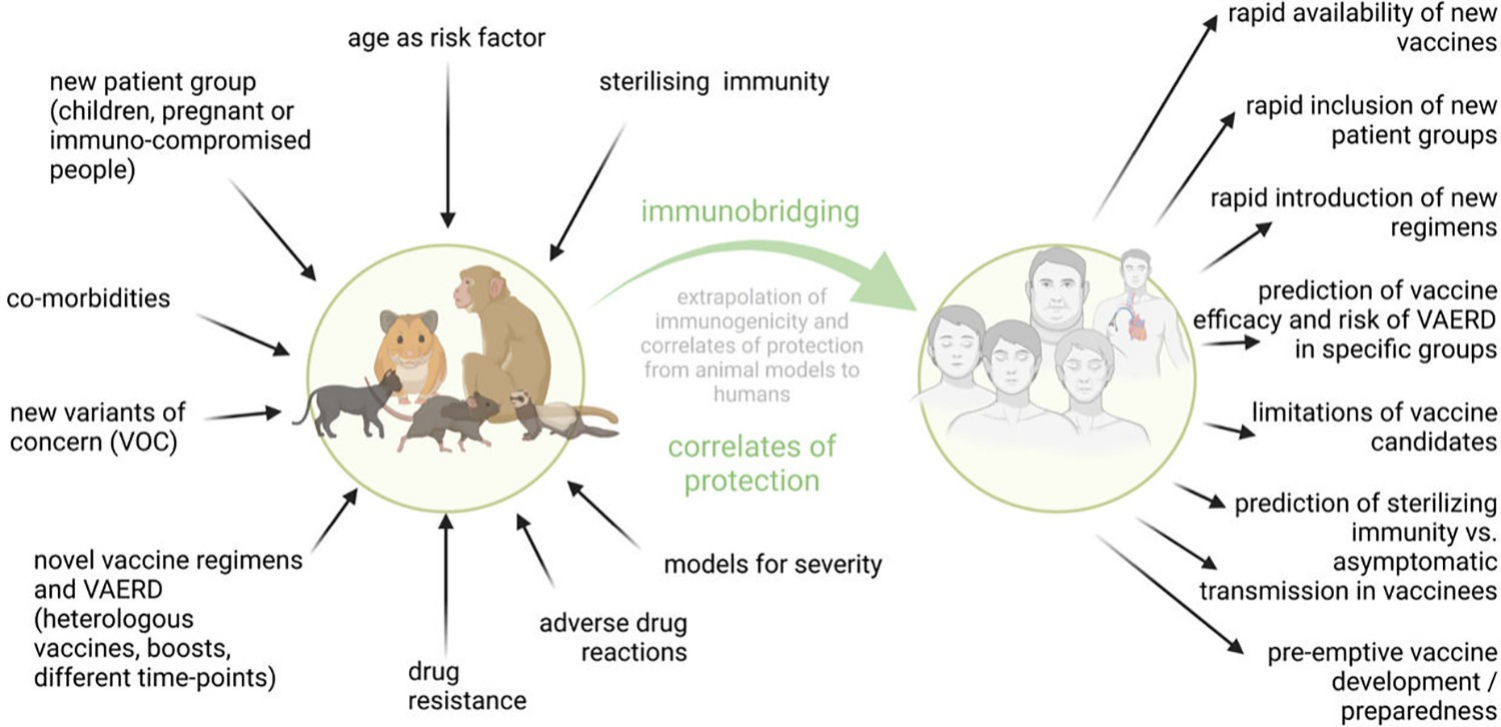
Animal models and immunobridging. Comparative and standardized studies in animal models such as those performed by WHO-COM scientists can help to extrapolate vaccine immunogenicity data across preclinical and clinical studies. Figure created with Biorender (Biorender.com). VAERD, vaccine-associated enhanced respiratory disease; VOC, variant of concern; WHO-COM, WHO COVID-19 Modeling group.

Establishing the immunological correlates of protection remains a key question for vaccine deployment and evaluation. While high levels of neutralizing antibodies in sera likely are associated with protection against disease, the contribution of cellular immunity to protection, including T cells and Fc-dependent effector antibody functions, remains uncertain. In fact, in Phase III SARS-CoV-2 mRNA and Ad26 vaccine clinical trials, protection against clinical infection could be seen even before the appearance of protective antibody titers in sera [89]. Moreover, correlates of protection against clinical disease and against asymptomatic infection and transmission likely differ. The establishment of robust correlates of protection is necessary to allow immunobridging, which extrapolates vaccine immunogenicity in humans to a protective effect, based on the immunogenicity and protection observed in animal models. Immunobridging will benefit from standardized immunological assays to quantify correlates of protection across animal experiments and clinical studies (Fig 2). It has been used for the expansion of mRNA vaccine approval to younger age groups [90] and will likely expedite the approval of second-generation vaccines without costly and lengthy efficacy studies. It will also help in determining whether and when boosting is needed, and whether vaccine strain updates are needed. One caveat might be that different vaccines might have different correlates of protection, making a universal correlate of protection difficult to achieve. For example, from NHP studies, it has been suggested that T cells can contribute to protection when antibody levels are suboptimal, or start to wane [91]. This indicates that correlates for long-term protection for an individual may change over time or may include multiple immune parameters. Therefore, it will be important to define immune thresholds for protection. These thresholds can be different for different target groups (the very young, the elderly, pregnant, immune-compromised, or people with comorbidities), which is currently being investigated in the available animal models that reflect the physiological and immunological status of these target groups. Meta-analyses of data generated in clinical trials and postapproval are crucial to validate the correlates of protection identified in preclinical studies.

Animal models also might be valuable to validate correlates of protection from infection, disease, and transmission. Passive antibody studies in multiple animal models have demonstrated that high titers of polyclonal or monoclonal neutralizing antibodies can protect from disease. Similarly, T cell depletion experiments and adoptive transfer experiments analogously can address the contribution of T cell immunity in protection from disease in mice and NHPs.

## Conclusions and future course

Animal models for SARS-CoV-2 infection have fostered the development of COVID-19 vaccines and therapeutics during the first year of the pandemic, several of which have been deployed on a global scale. Going forward, animal models can still fill important knowledge gaps. For example, preclinical animal studies will be important to understand disease progression and identify biomarkers that can aid us better predict the course of human disease. Animal model studies likely will allow the experimental validation of predicted correlates of protective or dysregulated immunity in humans. Similarly, animal models will be essential to evaluate VOC pathogenicity and transmissibility and to further assess the potential risk of VAERD, especially in the context of heterologous vaccination regimens. It will also be essential to develop SARS-CoV-2 animal infection models in which the virus replicates for extended periods of time, thus allowing for assessment of emergence of resistant variants against vaccines or therapies. Finally, as there is still much to learn about the role of comorbidities in human COVID-19, animal models with comorbidities will be needed to dissect the role of infection versus comorbidity in disease severity. These efforts may also lead to the design and evaluation of specific therapies against severe COVID-19 that function best in the background of particular medical conditions.
